# Dietary Intake of *trans* Fatty Acids in the Slovenian Population

**DOI:** 10.3390/nu13010207

**Published:** 2021-01-12

**Authors:** Nina Zupanič, Maša Hribar, Hristo Hristov, Živa Lavriša, Anita Kušar, Matej Gregorič, Urška Blaznik, Barbara Koroušić Seljak, Petra Golja, Rajko Vidrih, Katja Žmitek, Igor Pravst

**Affiliations:** 1 Nutrition Institute, Tržaška cesta 40, SI 1000 Ljubljana, Slovenia; nina.zupanic@nutris.org (N.Z.); masa.hribar@nutris.org (M.H.); hristo.hristov@nutris.org (H.H.); ziva.lavrisa@nutris.org (Ž.L.); anita.kusar@nutris.org (A.K.); igor.pravst@nutris.org (I.P.); 2 Jožef Stefan Institute, SI 1000 Ljubljana, Slovenia; barbara.korousic@ijs.si; 3 National Institute of Public Health, Trubarjeva 2, SI 1000 Ljubljana, Slovenia; matej.gregoric@nijz.si (M.G.); urska.blaznik@nijz.si (U.B.); 4 Biotechnical Faculty, University of Ljubljana, Jamnikarjeva 101, SI 1000 Ljubljana, Slovenia; petra.golja@bf.uni-lj.si (P.G.); rajko.vidrih@bf.uni-lj.si (R.V.); 5 VIST–Higher School of Applied Sciences, Gerbičeva cesta 51A, SI 1000 Ljubljana, Slovenia

**Keywords:** *trans* fatty acids, partially hydrogenated oils, dietary intake, 24-h recall, EU Menu, Slovenian population

## Abstract

Consumption of *trans* fatty acids (TFAs) has been unequivocally linked to several adverse health effects, with the increased risk of cardiovascular disease being one of the most well understood. To reduce TFA-related morbidity and mortality, several countries have imposed voluntary or mandatory measures to minimize the content of industrial TFAs (iTFAs) in the food supply. In 2018, Slovenia introduced a ban on iTFAs on top of preceding voluntary calls to industry to reduce its use of partially hydrogenated oils (PHOs) as the main source of iTFAs. To investigate the consumption of TFAs, data available from the nationally representative dietary survey SI.Menu were analyzed. The survey consisted of two 24-h non-consecutive day recalls from 1248 study participants from three age groups (10–17, 18–64, 65–74 years old), combined with socio-demographic, socio-economic, and lifestyle parameters. The analyses demonstrated that, on average, TFAs accounted for 0.38–0.50% of total energy intake (TEI). However, 13% of adolescents, 29.4% of adults, and 41.8% of the elderly population still consumed more than 0.50% TEI with TFAs. The main sources of TFAs in the diet were naturally present TFAs from butter, meat dishes, and meat products, regardless of the age group. Results indicate that following the reformulation activities, the major sources of TFAs in the diets of the Slovenian population now represent foods which are natural sources of TFAs.

## 1. Introduction

*Trans* fatty acids (TFAs) are fatty acid isomers with one or more double bonds in *trans* instead of *cis* configuration. TFAs can be produced in the process of industrial partial hydrogenation of unsaturated fats (i.e., vegetable oils), which was the previously used method for production of margarine and shortening, or can occur naturally as a result of bacterial biohydrogenation of unsaturated fatty acids in rumen and can therefore be found in meat, milk, and dairy products from ruminants [1].

The intake of industrially produced TFAs (iTFAs) has been found to negatively influence blood cholesterol profile, increase triglycerides [2], stimulate inflammatory responses [3], and increase mortality, particularly from coronary heart disease (CHD) [4]. According to a meta-analysis of four prospective cohort studies, with every 2% of total daily energy gained from TFAs, coronary heart disease incidence increases by 23% [1]. A substantial body of scientific evidence on adverse health effects of iTFAs has led public health organizations to establish a recommendation of an upper tolerable limit for TFA intake at 1% of total energy intake (TEI), although, according to Ref. [5], “the intake should be as low as possible”. The intake of TFAs has also been recognized as a key risk parameter in the Global Burden of Disease Study [6], in which optimal daily TFA intake (taking into account TFAs from all sources) was set at 0.5% of TEI or below. Moreover, the WHO’s action package called “REPLACE”, released in 2018, aimed to completely eliminate iTFAs from the global food supply by 2023 [7].

As one of the first countries to recognize iTFAs as a major public health threat, Denmark set a maximum limit of 2 g TFAs per 100 g of total fat in foods in 2004 [8]. The policy successfully reduced the amount of iTFAs present in the food supply, while, as a result of the action, the cardiovascular mortality rate also declined sharply [9]. By the end of 2018, similar legislation was introduced in 22 other countries, including Switzerland, Austria, Iceland, Hungary, and Norway [10,11]. In Slovenia, a ban on TFAs was introduced in March 2018 with the transitional period of one year [12], although the use of partially hydrogenated oils (PHOs) in Slovenian food supply had already decreased substantially between 2015 and 2017 [13]. During this period, TFAs were a subject of numerous media reports, putting pressure on the food industry to reformulate products containing PHOs [13]. In 2017, the only remaining categories of pre-packaged foods with a notable proportion of items containing PHOs were cakes, muffins, pastries, and biscuits [13]. Expected intake of iTFAs in the general population was therefore low, except for specific population groups, such as regular consumers of cakes, muffins, pastries, and biscuits of certain brands, which were high in iTFAs.

In 2010, reported estimated intakes of TFAs varied from 0.2 to 6.5% TEI across different countries worldwide, with the intakes being higher at younger ages. The estimated mean intake of total TFAs in Slovenian adults was approximately 1% TEI [14]. The objective of our study was to access current mean daily TFA intake levels using data collected within the Slovenian national dietary survey SI.Menu 2017/2018, and to identify major sources of TFAs in people’s diets.

## 2. Material and Methods

### 2.1. Study Design and Subjects

The data on food intake was obtained during the cross-sectional Slovenian national food consumption survey, SI.Menu 2017/2018, between March 2017 and April 2018, following the European Food Safety Authority (EFSA) Guidance on European Union (EU) Menu Methodology [15]. Methodology and sample characteristics are described in detail elsewhere [16]. In short, 2280 Slovenian residents aged 10–74 years were selected randomly, using the Central Register of Population of Slovenia. All selected participants received an invitation letter with all the information regarding the study and were later visited by the study interviewers who confirmed the eligibility of the participants and collected information from the respondents by interviews. A total of 62.2% of all invited participants joined the survey.

The study design was approved by the National Medical Ethics Committee (KME 53/07/16; approval No. 0120-337/2016 issued on 19.7.2016). Before enrolment in the survey, all participants were asked to sign written informed consent. If a participant was 18 years of age or younger, written consent was signed by the parent(s) or legal guardian(s).

### 2.2. Food Consumption Data

#### 2.2.1. General Questionnaire and Anthropometric Measurements

During the first interviewer’s visit, the participants filled in a general questionnaire, which assessed general socio-demographic and socio-economic determinants such as number of household members, marital status, level of education, monthly net income of the household, as well as habitual frequency and duration of physical activity. For the purpose of this study, the latter was subsequently converted into the International Physical Activity Questionnaire (IPAQ) score [17]. During the first interview, participants’ body mass and body height were assessed by the interviewer using portable calibrated scales. Body mass index (BMI; kg/m^2^) was determined with the cut-off point for overweightness set at 25 kg/m^2^, except for adolescents, where gender/age adjusted cut-off points (>1SD) were applied [18,19].

#### 2.2.2. 24-h Dietary Recalls

Assessment of dietary intake was performed using a two 24-h dietary recall method. The two recalls were carried out on the same day of the week one to three weeks apart and took place at the participant’s home. To obtain a sample that would take into consideration the variations in dietary habits between working and weekend days, 71% of the recalls were performed on work days and 29% on weekends. Altogether, 87% of the recalls were repeated within 7 days after the first recall, while the rest were completed within the next two weeks. The recall was structured to follow a daily meal timeline to help the participants systematically recollect foods and beverages consumed during the previous day. To better estimate the portion sizes of reported foods, a nationally adjusted booklet containing 46 pictures of different food products or simple recipes was developed especially for this purpose. Each food product was presented in 6 different portion sizes to help interviewers and participants determine the quantity of the ingested dish. A picture book was validated according to the method of conceptualization in May 2015 at Biotechnical Faculty, University of Ljubljana, Slovenia [16].

Data from the recalls were collected using an application that was based on the Open Platform for Clinical Nutrition (OPEN), developed by the Jozef Stefan Institute (Ljubljana, Slovenia) for the purpose of SI.Menu survey. The OPEN app is an extension of the Slovenian food composition database [20], which includes data on generic and branded foods and provides a list of ingredients for traditional and other recipes frequently consumed in Slovenia. Missing data were completed with data from other European (EuroFIR) and United States Department of Agriculture (USDA) food composition databases [21].

### 2.3. Assessment of TFA Content

Energy and nutrient content of all reported foods and beverages was calculated based on the data available on the national platform for clinical nutrition, called the Open Platform for Clinical Nutrition (OPEN) [20]. Missing data for TFA content were extracted from the previously established database [22], which was compiled by analytical assessment of foods from Slovenian food. In the OPEN database, the energy value of foods and beverages is calculated based on macronutrients, alcohol, and dietary fiber content, using an approach provided in the EU Regulation 1169/2011 on the provision of food information to consumers [23]. When information on dietary fiber content was available, total energy value was calculated as total available energy using established conversion factors for the calculation of energy (i.e., 17 kJ per g of digestible carbohydrates and protein, 37 kJ per g of fat, and 8 kJ per g of dietary fiber) [23]. In certain foods and beverages, in which a very low content of dietary fiber was expected, total energy content was calculated as 17 kJ per g of total carbohydrates. To enable accurate nutrient profile formation for more complex foods and dishes, a disaggregation method was applied based on the recipes provided by the subjects, when applicable, or traditional recipes collected in OPKP, considering both the yield and retention factors [20]. To differentiate between pre-packaged and non-packaged foods, each food/beverage item was assigned as branded or non-branded. For the purpose of the statistical analyses in this study, each reported food was allotted to one of 17 predefined categories, which were further divided into 96 subcategories. The categorization system was adapted from Dunford et al. [24], with the additional subcategories added only for non-packaged and home-cooked foods.

### 2.4. Final Sample for Data Analyses

Inclusion and exclusion criteria as well as exclusion of under- and over-reporters in SI.Menu study were previously described [25]. In short, 97 participants were excluded before the final analyses due to incomplete anthropometric data (*n* = 12), incomplete 24-h recall data (*n* = 36), or under- and over-reporting (*n* = 49). The final sample consisted of 1248 subjects: 468 adolescents (mean age 13.4 ± 2.37), 364 adults (mean age 43.6 ± 13.81), and 416 elderly (mean age 68.7 ± 2.7). Under- and over-reporting cut-off points were calculated by Goldberg method [26], considering adaption by Black et al. [27]. Basic metabolic rate (BMR) was calculated based on gender, age, body height, and body mass using the method described by Harris et al. [28] and adapted by Roza and Shizgal [29].

### 2.5. Statistical Analyses

All analyzed participants completed both 24-h recalls and provided answers to the survey questionnaire. The energy- and TFA-intake estimates for each of the two recalls and per each of the three age categories were normalized using log transformation method. The TFA estimates were further energy adjusted using nutrient residual method [30]. As the 24h recalls measured only short-term consumption patterns, both recalls were combined to estimate the habitual TFA consumption using the multiple source method (MSM) [31]. The algorithm of the MSM method was used to estimate an average TFA intake adjusted for interpersonal variance under the assumption that all survey participants are habitual consumers of TFAs. The data referring to the national representative sample was weighted using the iterative proportional fitting method [32] based on the census data from 2017 to produce representative results according to age, gender, and region of living.

Descriptive statistics for age cohorts and for different socio-demographic-, anthropometric-, and individual-based variables within each age group are shown as frequencies and proportions, or medians and means with standard deviations (SD). Multiple linear and logistic regression models for all three age cohorts were undertaken separately to assess the significant differences between different sub-populations in terms of TFA consumption based on TEI. The unadjusted means of TFA in % TEI were determined by gender, region, BMI, and IPAQ levels for all age groups, education, income for adults and elderly, and employment status for adults only. The prevalence of consumption of more than 0.5% TEI in TFAs was adjusted for socio-demographic, anthropometric, and lifestyle parameters. The logistic regression analysis was used to determine independent predictors for TFA intake greater than 0.5% of TEI with a maximum likelihood as the estimation method for the model parameters. Odds ratios (ORs) with 95% confidence intervals (CIs) were used as a measure of association with exposure to more than 0.5% TEI from TFA. Statistical significance was set at *p* < 0.05. All analyses were performed using STATA version 15.1 (StataCorp LLC, Coledge Station, TX, USA).

## 3. Results

**Figure 1:**
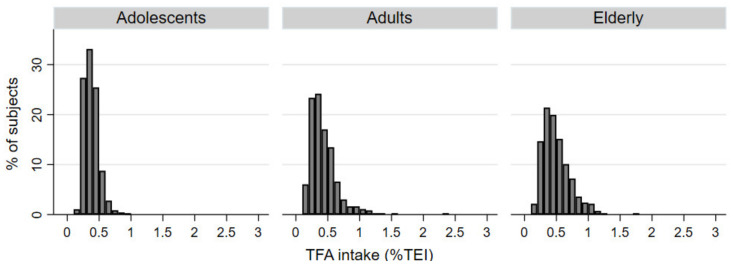
Histograms of daily dietary intake of total *trans* fatty acids (TFAs) expressed in percentage of total energy intake (%TEI) in adolescents, adults, and elderly populations.

Considering the study sampling design, we estimated dietary intakes of TFAs in the Slovenian population separately for adolescents, adults, and the elderly (Table 1). Estimates were done for both the amount of consumed TFAs daily (0.68 g, 0.77 g, and 0.89 g, respectively) and for total energy intake (TEI) from TFAs (0.38–0.50%), which is particularly relevant and was therefore used for statistical analyses and modelling. In adolescents, the TFAs represented on average 0.38% (CI: 0.35–0.39) of TEI. The percentage was marginally higher in adults, where 0.42% (CI: 0.40–0.45) of TEI came from TFAs and was the highest in the elderly, whose consumption of TFAs accounted for 0.5% (CI: 0.47–0.53) of TEI (Table 1, Figure 1).

On average, women consumed significantly higher % of TEI with TFAs compared to men, regardless of their age. A significant difference was observed in adults with different BMI, with higher TFA consumption recorded in overweight and obese individuals, as compared to those with normal BMI. In the adjusted analysis, there were no other significant differences in TFA consumption in other socio-demographic variables, including region, education, family net income, IPAQ score, or employment status (Table 2).

Additional analyses demonstrated that 13% adolescents, 29.4% adults, and 41.8% of the elderly population consume more than 0.5% TEI with TFAs. Odds for exceeding 0.5% TEI coming from TFAs were significantly higher among adolescent girls and elderly women compared to men. Although not significant, a notable gender-related pattern was observed among adults as well. None of the remaining variables significantly influenced the prevalence exceeding the 0.5% TEI limit (Table 3).

**Figure 2:**
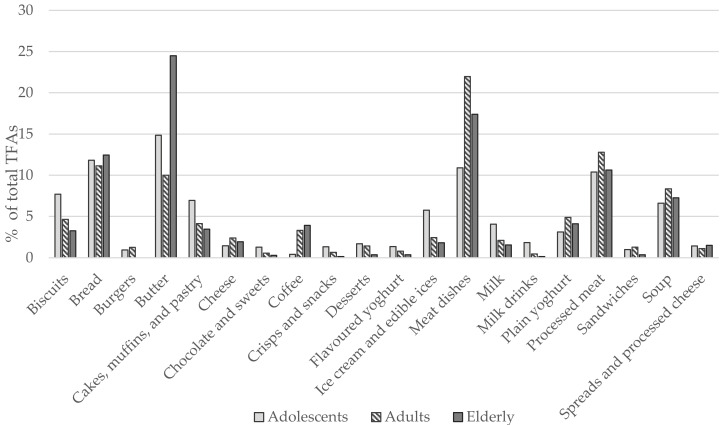
Relative contribution of food categories to *trans* fatty acid (TFA) intake among different age groups (% of total TFAs intake). Note: “Coffee” category also includes coffee drinks that can contain milk or cream.

Major sources of TFAs among Slovenian adolescents were butter (14.8%), bread (11.8%), meat dishes (10.9%), and processed meat (10.4%). Biscuits (7.7%), cakes, muffins, and pastry (6.9%), soups (6.6%), and ice cream and edible ices (5.8%), were also notable sources of TFAs among the youth. The majority of TFA intake among adults came from meat dishes (22%) and processed meat (12.8%), but also bread (11.1%), butter (10%), and soups (8.3%). In the elderly, butter was the predominant source of TFAs (24.5%), followed by meat dishes (17.4%), bread (12.4%), processed meat (10.6%), and soups (7.3%) (Figure 2).

## 4. Discussion

With an average value between 0.38 and 0.50% TEI, total intake of TFAs in Slovenia in 2017/2018 was much lower than the estimate of Micha et al. for 2010 [14] and also lower than WHO recommendations [5] of up to 1% TEI. Worldwide analyses of dietary risks among 195 countries demonstrated that in 2017, TFA intake in Central and Western European countries averaged at around 0.4% TEI [6], which is comparable with the TFAs intakes estimated in our study. Much higher intakes were, on the other hand, reported from high-income North America and certain Latin American countries [6].

Several studies have reported a decreasing consumption of TFAs during the last decades due to active public campaigns as well as voluntary or mandatory measures following the cumulative evidence on the adverse health effects of iTFA consumption [33,34,35]. However, in 2016, Stender and colleagues [36] published a market basket investigation, in which they reported a 3-fold increase in the availability of biscuits containing PHOs in Slovenia between 2012 and 2014. Our subsequent studies demonstrated that by 2017, the proportion of pre-packaged foods containing PHOs had already dropped significantly [13]. The highest proportion of TFA-containing items was found among biscuits, for which additional analyses demonstrated that they would exceed EU regulatory TFA limits in 69% of cases among PHO-containing products [22]. However, the present study revealed that biscuits account only for approximately 7% of all TFAs consumed, which can be either of industrial or natural sources.

Even though we were unable to differentiate between industrially produced and naturally present TFAs, due to limited data about the food composition, relative contribution of different food categories demonstrated that a great proportion of TFAs in Slovenia were consumed in the form of vaccenic acid, a natural TFA found in dairy and beef products. While some evidence suggests that both industrial and ruminant TFAs show similar effects on composition of plasma lipoproteins [2,37], the topic remains controversial [38,39,40,41,42]. Their chemical structure is indeed different and some evidence suggests that compared to ruminant vaccenic acid, iTFAs have much higher potency for promoting inflammation, endoplasmic reticulum stress, and cholesterol synthesis [43]. Nevertheless, the intake of TFAs of animal origin are generally well below the recommended 1% TEI, which greatly limits their possible impact on cardiovascular disease risk. The major sources of naturally present TFAs in Slovenia were butter, meat dishes, and processed meat, which jointly contributed on average 36% of all TFAs consumed among adolescents and up to 53% in the elderly. Especially due to high butter consumption, the intake of TFAs is the highest among elderly Slovenians, which is in contrast to the findings of Micha and colleagues [14], whose systematic analysis showed TFA intake tended to be higher among younger participants. The observed discrepancy most likely results from a shift from artificial towards predominantly natural dietary sources of TFAs, due to the diminished use of PHOs. Adolescents tend to consume more biscuits and other foods which used to be high in iTFAs, while adults and the elderly consume more meat and butter, which now represent the predominant sources of TFAs.

Successful elimination of iTFAs from the food supply is a significant advancement in cardiovascular disease prevention, although concerns have been voiced whether TFAs in processed foods might not necessarily be replaced by healthier alternatives, such as mono- and polyunsaturated vegetable oils [44,45]. Replacement of iTFAs with another solid fat could concomitantly increase the intake of saturated fats, which would hamper the efforts to lower their intake. However, studies from the U.S. and Canada showed that a decrease in TFA content in the food supply was not accompanied by an increase in saturated fat content [46,47]. Whether similar trends took place in Europe and other parts of the world has not been investigated yet. Furthermore, an overview of identified food sources of TFAs in the Slovenian population highlighted that further reduction of TFA intake would be only possible with reduced intake of foods, which are natural sources of TFAs, particularly meat, butter and dairy products. Regarding this is should be noted, that some evidence also suggests some possible beneficial effects of specific types of TFAs, which are naturally present in these foods, such as *trans* vaccenic acid [38,39,40,41,42].

The strengths of this study are in the nationally representative sample for Slovenia (aged 10–74 years), the use of a robust methodological approach, and the fact that the actual data about TFA levels for many foods were available, as they had been collected within the “Trans fats in foods” project [48]. An additional strength of the present study is that we did not only estimate TFAs intakes but also investigated lifestyle and socio-demographic parameters, which might be associated with TFA intakes. However, there are also some important limitations that should be noted. Namely, data collection with two 24 h recalls can result in misreporting and has limited accuracy [49], but other methodological approaches also have considerable limitations. To minimize errors, interviewers were trained to have participants recollect all ingested foods and drinks and corresponding portion sizes as accurately as possible. The remaining under- and over-reporting errors were corrected for during data analyses. Alternatively, using blood biomarkers of TFA plasma levels could increase the precision of TFA intake estimates in the population, but such an approach would not reveal the major dietary sources of TFAs. A limitation of the present study is the quantification of only total TFA intake, due to difficult distinctions between industrial and naturally present TFAs in complex food products. However, the reported adverse health effects tend to be similar for both TFA sources [50] and therefore the used approach should be equally relevant. Another limitation comes from assessing TFA content in foods. Relying on data from food composition databases can introduce errors, as the data can be incomplete, especially when it comes to specific food constituents, such as TFAs. To decrease the magnitude of error, products known to contain the largest amounts of iTFAs had had their TFA content determined analytically in one of our preceding studies [22]. We should also note that the used methodological approach does not enable us to evaluate variability in food consumption and identification of specific risk scenarios, such as TFA intakes in brand-loyal consumers of PHO-containing biscuits and cakes, for which the probabilistic exposure assessment would be an appropriate method.

## 5. Conclusions

In last decade, the intake of TFAs in Slovenia has dropped under 0.5% of TEI, with butter and meat products becoming a predominant source of TFAs consumed. With additional mandatory restrictions on the use of PHOs in foods in place, more vulnerable specific consumer groups are now also protected. Further reduction of TFA intakes would be only possible with considerable changes in dietary patterns towards less full-fat dairy and high-fat meats, however, a complete elimination of TFAs is not feasible within the scope of a balanced omnivorous diet.

## Figures and Tables

**Table 1:** Population-weighted *trans* fatty acid (TFA) intake (g/day) and prevalence of total energy intake (TEI) from TFAs > 0.5%/1.0% daily (95% CI), both cumulative and according to gender.

	Adolescents (10–17 years)			Adults (18–64 years)			Elderly (65–74 years)		
	All	Male	Female	All	Male	Female	All	Male	Female
Sample Size									
N (%)	468 (100)	238 (50.85)	230 (49.15)	364 (100)	173 (47.53)	191(52.47)	416 (100)	213 (51.20)	203 (48.80)
Margin of error (%)	4.53	6.36	6.47	5.14	7.45	7.09	4.81	6.71	6.88
TFAs intake									
Mean [g/day]	0.68	0.68	0.68	0.77	0.78	0.75	0.89	0.90	0.87
Median [g/day]	0.67	0.67	0.68	0.73	0.75	0.72	0.84	0.86	0.82
Mean % TEI (95% CI)	0.38 (0.35–0.39)	0.33 (0.31–0.35)	0.41 (0.38–0.45)	0.42 (0.40–0.45)	0.38 (0.35–0.41)	0.46 (0.43–0.50)	0.50 (0.47–0.53)	0.48 (0.43–0.52)	0.51 (0.47–0.56)
TEI from TFAs (%)									
TFA > 0.5% TEI (95% CI)	11.50 (8.34–15.7)	7.5 (4.6–12.2)	15.7 (10.5–23.0)	28.9 (23.1–33.2)	23.0 (16.8–30.5)	32.8 (25.9–40.6)	43.9 (34.1–54.1)	41.6 (24.8–60.6)	45.9 (35.9–56.3)
TFA > 1.0% TEI (95% CI)				2.51 (1.25–4.96)	1.01 (2.47–4.06)	4.02 (1.82–8.66)	3.02 (1.64–5.50)	4.18 (1.89–9.00)	1.96 (0.74–5.14)

Notes: 95% CI: 95% confidence interval; % TEI: percentage of total energy intake; TFA > 1.0% TEI, percentage of participants not adhering to WHO recommendation for intake of TFAs < 1.0% of TEI; TFA > 0.5% TEI, percentage of participants not adhering to Global Burden of Disease Study target value for intake of TFAs < 0.5% TEI.

**Table 2:** Adjusted mean (95% CI) levels of *trans* fatty acid (TFA; % TEI) intake by gender, region, body mass index (BMI), international physical activity questionnaire (IPAQ) score, education, income, and employment for different age groups.

Variable		Adolescents (10–17 years)		Adults (18–64 years)		Elderly (65–74 years)	
		*n* (%)	Adjusted	*n* (%)	Adjusted	*n* (%)	Adjusted
Overall		468 (37.5)		364 (29.2)		416 (33.3)	
Sex	Male	238 (50.9)	0.35 (0.33–0.36)	173 (47.5)	0.39 (0.35–0.42)	213 (51.2)	0.46 (0.43–0.49)
	Female	230 (49.1)	0.41 (0.40–0.43)	191 (52.5)	0.47 (0.44–0.51)	203 (48.8)	0.54 (0.50–0.57)
Place of living	Rural	270 (57.7)	0.37 (0.37–0.39)	202 (55.5)	0.41 (0.38–0.45)	229 (55.1)	0.48 (0.45–0.51)
	Intermediate	76 (16.2)	0.37 (0.34–0.39)	56 (15.4)	0.47 (0.40–0.53)	71 (17.1)	0.51 (0.46–0.56)
	Urban	122 (26.1)	0.40 (0.38–0.42)	106 (29.1)	0.45 (0.40–0.49)	116 (27.9)	0.52 (0.48–0.57)
Education	No university degree	-	-	249 (68.4)	0.44 (0.41–0.47)	342 (82.2)	0.50 (0.48–0.53)
	University degree			115 (31.6)	0.42 (0.38–0.47)	74 (17.8)	0.48 (0.43–0.54)
Family net income	Below average	-	-	118 (38.4)	0.44 (0.40–0.48)	269 (71.5)	0.51 (0.48–0.54)
	Above average			189 (61.6)	0.43 (0.40–0.46)	107 (28.5)	0.47 (0.43–0.51)
BMI	Normal	301 (64.3)	0.37 (0.36–0.38)	148 (40.7)	0.40 (0.36–0.44)	108 (26.0)	0.51 (0.47–0.55)
	Overweight/obese	167 (35.7)	0.39 (0.38–0.41)	216 (59.3)	0.46 (0.42–0.49)	308 (74.0)	0.50 (0.47–0.52)
IPAQ	Low intensity	108 (23.3)	0.37 (0.35–0.39)	127 (35.3)	0.42 (0.38–0.46)	137 (33.4)	0.49 (0.46–0.53)
	Moderate	141 (30.5)	0.39 (0.38–0.41)	108 (30.0)	0.45(0.41–0.50)	133 (32.4)	0.52 (0.48–0.55)
	High intensity	214 (46.2)	0.38 (0.36–0.39)	125 (34.7)	0.44 (0.39–0.48)	140 (34.2)	0.49 (0.45–0.52)
Employment	Employed	-	-	226 (62.1)	0.42 (0.39–0.45)	-	-
	Unemployed			42 (11.5)	0.40 (0.33–0.48)		
	Student			32 (8.8)	0.43 (0.34–0.52)		
	Retired			64 (17.6)	0.50 (0.44–0.56)		

Note: Body mass index (BMI) was considered as normal below 25 kg/m^2^, except for adolescents, where gender/age adjusted cut-off points [18,19] were used. Linear regression analysis conducted on samples with excluded missing values (family net income: *n* = 57 (adults) and 40 (elderly); IPAQ: *n* = 5 (adolescents), 4 (adults), 6 (elderly)); identified predictors accounting for difference in the %TEI from TFA: *p* < 0.001 sex (adolescents), *p* < 0.001 sex (adults), *p* = 0.0402 BMI (adults), *p* < 0.001 sex (elderly).

**Table 3:** Percentage of the population exceeding 0.5% of total energy intake (TEI) from *trans* fatty acid (TFA) intake by sex, place of living, education, family net income, body mass index (BMI), international physical activity questionnaire (IPAQ) score, and employment.

Variable		Adolescents (10–17 Years Old)			Adults (18–64 Years Old)			Elderly (65–74 Years Old)		
		*n*	>0.5% TEI *n* (%)	Odds Ratio *	*n*	>0.5% TEI *n* (%)	Odds Ratio *	*n*	>0.5% TEI *n* (%)	Odds Ratio *
Overall		468	61 (13.03)		364	107 (29.40)		416	174 (41.83)	
Sex	Male	238	20 (8.40)	1	173	42 (24.28)	1	213	64 (30.05)	1
	Female	230	41 (17.83)	2.45 (1.35–4.46)	191	65 (34.03)	1.65 (0.98–2.78)	203	110 (54.19)	2.45 (1.58–3.81)
Place of living	Rural	270	33 (12.22)	1	202	53 (26.24)	1	229	84 (36.68)	1
	Intermediate	76	6 (7.89)	0.65 (0.25–1.64)	56	19 (33.93)	1.48 (0.73–2.98)	71	31 (43.66)	1.06 (0.59–1.92)
	Urban	122	22 (18.03)	1.71 (0.92–3.15)	106	35 (33.02)	1.30 (0.74–2.30)	116	59 (50.86)	1.68 (1.01–2.80)
Education	No university degree		-	-	249	75 (30.12)	1	342	146 (42.69)	1
	University degree				115	32 (27.83)	0.96 (0.53–1.75)	74	28 (37.84)	0.66 (0.36–1.24)
Family net income	Below average (≤1300 €)		-	-	118	39 (33.05)	1	269	111 (41.26)	1
	Above average (>1300 €)				189	56 (29.63)	1.03 (0.58–1.85)	107	42 (39.25)	0.96 (0.58–1.60)
BMI	Normal	301	33(10.96)	1	148	38 (25.68)	1	108	55 (50.93)	1
	Overweight (including obese)	167	28 (16.77)	1.64 (0.92–2.91)	216	69 (31.94)	1.66 (0.95–2.90)	308	119 (38.64)	0.75 (0.46–1.24)
IPAQ	Low intensity	108	13 (12.04)	1	127	31 (24.41)	1	137	53 (38.69)	1
	Moderate	141	25 (17.73)	1.28 (0.61–2.70)	108	34 (31.48)	1.53 (0.82–2.87)	133	60 (45.11)	1.12 (0.66–1.91)
	High intensity	214	20 (9.35)	0.71 (0.34–1.53)	125	41 (32.80)	1.37 (0.73–2.55)	140	60 (42.86)	1.13 (0.66–1.91)
Employment	Employed		-	-	226	61 (26.99)	1		-	-
	Unemployed				42	13 (30.95)	0.97 (0.40–2.33)			
	Student				32	8 (25.00)	1.51 (0.55–4.13)			
	Retired				64	25 (39.06)	1.98 (0.97–4.02)			

Note: Body mass index (BMI) was considered as normal below 25 kg/m^2^, except for adolescents, where gender/age adjusted cut-off points [18,19] were used. Logistic regression analysis conducted on samples with excluded missing values (family net income: *n* = 57 (adults) and 40 (elderly); IPAQ: *n* = 5 (adolescents), 4 (adults), 6 (elderly)). * Odds ratio for exceeding 0.5% TEI from TFA intake; identified predictors accounting for >0.5% TEI from TFA: *p* = 0.0026 sex (adolescents), *p* = 0.0543 sex (adults), *p* < 0.0001 sex (elderly).

## Data Availability

The data presented in this study are available on request from the corresponding author.
